# Molecular Cloning and Sequence Analysis of a Phenylalanine Ammonia-Lyase Gene from *Dendrobium*

**DOI:** 10.1371/journal.pone.0062352

**Published:** 2013-04-30

**Authors:** Qing Jin, Yao Yao, Yongping Cai, Yi Lin

**Affiliations:** Anhui Agricultural University, Hefei, China; University of South Florida, United States of America

## Abstract

In this study, a phenylalanine ammonia-lyase (PAL) gene was cloned from *Dendrobium candidum* using homology cloning and RACE. The full-length sequence and catalytic active sites that appear in PAL proteins of *Arabidopsis thaliana* and *Nicotiana tabacum* are also found: PAL cDNA of *D. candidum* (designated Dc-PAL1, GenBank No. JQ765748) has 2,458 bps and contains a complete open reading frame (ORF) of 2,142 bps, which encodes 713 amino acid residues. The amino acid sequence of DcPAL1 has more than 80% sequence identity with the PAL genes of other plants, as indicated by multiple alignments. The dominant sites and catalytic active sites, which are similar to that showing in PAL proteins of *Arabidopsis thaliana* and *Nicotiana tabacum*, are also found in DcPAL1. Phylogenetic tree analysis revealed that DcPAL is more closely related to PALs from orchidaceae plants than to those of other plants. The differential expression patterns of PAL in protocorm-like body, leaf, stem, and root, suggest that the PAL gene performs multiple physiological functions in *Dendrobium candidum*.

## Introduction

*Dendrobium* Sw. is a species of perennial herb in the Orchidaceae family, which was first introduced in the book *Shennong Bencao Jing* and is well-known for its valuable medical uses. Approximately 1,000 types of *Dendrobium* Sw. are widely distributed in tropical and subtropical regions of Asia, Europe, Oceania, and other areas. *Dendrobium* Sw. has been recognized as a medicinal herb because it can be used for maintaining gastric tonicity, enhancing the production of bodily fluids, and relieving symptoms such as dryness of the throat and thirst with blurred vision[1].

The officinal effect of *Dendrobium* Sw. is primarily produced by secondary metabolites[2]. As determined using modern pharmacological techniques, the main chemical compounds of *Dendrobium* Sw. include polysaccharides, alkaloids, and others [3], [4]. Alkaloids are the most important secondary metabolite of *Dendrobium* Sw., as it has many officinal effects, including anticancer, neuroprotective, anti-angiogenesis, and immunomodulatory[5]–[8].

Alkaloid synthesis is catalyzed by phenylalanine ammonia-lyase (PAL), a critical enzyme that controls the speed of the first step in the biosynthesis of phenylpropanoid metabolites, i.e., the non-oxidative deamination of phenylalanine to trans-cinnamic acid and ammonia[9], [10]. Phenylpropanoids produce many secondary metabolites in plants, such as flavonoids, plant hormones, anthocyanins, lignins, phytoalexins, and benzoic acid[11]. Research on PAL has always attracted a lot of attention, because PAL plays a key role in connecting plant primary metabolism and phenylpropanoid metabolism and is also involved in the biosynthesis of signaling molecules and salicylic acid[12], [13].

PAL exists in all higher plants and is also found in some fungi and cyanobacteria[14]. However, PAL has not yet been extracted from Eubacteria, Archaea, and animals. Previous research shows that PAL purified from French beans [15] and tomatoes[16] exists as a tetramer[17]. It is possible to produce functional heterotetrameric enzymes[18] by coexpressing different tobacco PAL proteins in *Escherichia coli* [19].

Recent research has indicated that PAL influences the biosynthesis of alkaloid in *Dendrobium* Sw. plants, and the activity of PAL increases with the synthesis of alkaloids. However, the influence of PAL on the growth of this plant has not yet been clearly elucidated. Indeed, it will be important to determine how PAL influences the growth of *Dendrobium* Sw. in terms of physiology and biochemistry. In addition, it will be important to analyze how PAL affects the production and control of secondary metabolic products in *Dendrobium* Sw., with the aim of overcoming the low rate of production of secondary metabolites. The aim of the current study was to identify and analyze the PAL gene and encoded protein in *Dendrobium* Sw.

## Materials and Methods

### Plant materials

Protocorm-like bodies from *Dendrobium candidum* were supplied by Anhui Agricultural University of China. Callus induction was performed according to the methods used for somatic embryogenesis. The growing calli were subcultured to induce somatic embryos on modified MS medium. Seedlings were grown in a growth chamber with a 12 h/12 h light/dark cycle, a 25°C/20°C day/night temperature, and a 450 µmol m^−2^s^−1^ light intensity. Leaves, stems, and roots were harvested directly into liquid nitrogen and stored at −80°C.

### RNA isolation and reverse transcription

Total RNA was extracted from different tissues of *Dendrobium candidum* using a Tiangen (RNAprep pure Plant Kit. TIANGEN BIOTECH BEIJING CO., LTD.) RNA extraction reagent followed the manufacturer's instructions. Spectrophotometry was then performed to measure the concentration of each total RNA sample.

Using a PrimScript™ first-strand cDNA synthesis kit (TAKARA BIOTECHNOLOGY DALIAN CO., LTD.), first-strand cDNA was synthesized by reverse transcription (RT) to transcribe poly (A)^+^mRNA with oligo-dT primers following the manufacturer's instructions. The cDNA was stored at −20°C for further analysis (RT-QPCR).

### Amplification of the PAL gene using the RACE method

To design and simplify the primers used in this study, which are shown in Table 1, the homology of the amino acid sequence was identified using DNAStar software by comparing the sequence with other amino acid sequences registered in the GenBank library, such as sequences from phalaenopsis, Lycoris radiata, and Cinnamomum osmophloeum. Total RNA extracted from Dendrobium candidum tissues was used as a template to amplify the Dc-PAL cDNA. Both 3′-RACE (3′-RACE System, Invitrogen, Carlsbad, CA, USA) and 5′-RACE (Smart Race Kit, Clontech, Palo Alto, CA, USA) were performed, following the manufacturers' instructions. Gene-specific primers of the Dc-PAL gene were designed using information from previously cloned fragments. First, AP (as the RT primer) and AUAP (as the universal amplification primer) were used for 3′-RACE. Two groups of three gene-specific primers, PAL-3GSP1, PAL-3GSP2, PAL-3GSP3 and PAL-5GSP1, PAL-5GSP2, PAL-5GSP3, were used for 3′-RACE and 5′-RACE, respectively. The primer UPM was used as the first amplification primer and NUP was used as the nested primer. Touchdown-PCR reactions were performed at 94°C (pre-denaturation) for 3 min, followed by 94°C for 30 s, 68°C for 30 s, and 72°C for 1 min in the first cycle, and the anneal temperature was decreased 1°C per cycle. After eleven cycles, the conditions were changed to 94°C for 30 s, 57°C for 30 s, and 72°C for 1 min for 19 cycles. The duration of the 72°C elongation step was 7 min.

**Table 1:** Primers used in this study.

Name	Sequence (5′-3′)
PAL-F1	AAYACNYTNYTNCARGG
PAL-F2	AARCAYCAYCCNGGNCARAT
PAL-F3	CARAARCCNAARCARGA
PAL-R1	TCYTGYTTNGGYTTYTG
PAL-R2	CCYTGRAARTTNCCNCCRTG
PAL-R3	ACRTCYTGRTTRTGYTGYTC
PAL-3GSP1	AACTTCCAGGGCACCCCCATCGGC
PAL-3GSP2	ATGGACAATACAAGGCTCGCCATTGCC
PAL-3GSP3	CAACAACGGTTTGCCATCCAATCTCTC
PAL-5GSP1	CTCCCTCTCAATAGACTTGGTTGCTGC
PAL-5GSP2	GAGGACCGAGCCATTGGGGTGAAGTGC
PAL-5GSP3	CATAGCGGTCCTGTTTCGGCTTCTGC
PAL-RTF	TGTGAAGAACACGGTGAGCC
PAL-RTR	TCGGCATAGGCAAGCACATA
β-actin-RTF	GGTATTGTGTTGGATTCCG
β-actin-RTR	TGAGTAGCCCCTCTCTGTGAG
AP	GGCCACGCGTCGACTAGTACTTTTTTTTTTTTTTTTT
AUAP	GGCCACGCGTCGACTAGTAC
UPM	Long:CTAATACGACTCACTATAGGGCAAGCAGTGGTAT-CAACGCAGAGTShort:CTAATACGACTCACTATAGGGC
NUP	AAGCAGTGGTATCAACGCAGAGT

### Subcloning and sequencing

The PCR fragments were then subjected to electrophoresis on a 1.5% agarose gel to compare the length differences between fragments. Amplified cDNA fragments were ligated to the pMD18-T vector (Catalog ID: D101A) following the manufacturer's instructions. Recombinant bacteria were selected by blue/white screening and verified by PCR. Nucleotide sequencing was then performed by Shanghai Sangon Biotech Company after obtaining DNA from cultured *E. coli*.

### Nucleotide sequence and bioinformatics analysis

The full-length cDNAs of *Dendrobium candidum* PAL were obtained using DNAStar software to splice the cloned gene fragments, which were then analyzed using a program that is available on the NCBI website (http://www.ncbi.nlm.nih.gov/). Searches for ORFs and prediction of nucleotide translation products were performed using the ORF Finder tool (http://www.ncbi.nlm.nih.gov/gorf/gorf.html). The fundamental properties and structural features of the proteins were predicted using a tool provided by ExPASy (http://www.expasy.org/). Alignments of multiple amino acid sequences were carried out using the Clustal W tool in the MEGA 3.1 program. A phylogenetic tree of the DcPAL gene was then produced with Clustal W (1.83). Furthermore, a Phylip distance matrix was generated with 2,000 bootstrap trials using MEGA 3.1. Phylogenetic relationships were deduced using the PDB database (http://www.rcsb.org/pdb/ho/) and the online tool SWISS-MODEL (http://swiss-model.expasy.org/).

### Analysis of the expression of the PAL gene

Tissue-specific expression of PAL was analyzed using RT-qPCR, which was performed by Biorad real-time fluorescence quantitative PCR. Three samples were collected from protocorm-like bodies, roots, stems, and leaves, and each sample was measured in duplicate. By using a Nucleotide quantitative detection instrument to test each RNA sample, it has been found that all the OD260/280 values are around 1.8 to 2.1. The qPCR amplification primers, (the primer's sequences are shown in Table 1, and the amplification product is 135 bp), were designed based on the gene sequence that was obtained. To amplify the gene fragments, dilute cDNA was used as a template in a 20 µL PCR reaction with 10 µL SYBR®Premix Ex Taq™ II (2×), 1 µL diluted cDNA, and 0.5 µL each of the primers PAL-RTF and PAL-RTR. The PCR reaction was performed as follows: 50°C for 2 min, followed by incubation for 30 s at 95°C and denaturation for 15 s at 95°C, annealing for 20 s at 60°C, and 40 cycles of elongation at 72°C for 20 s. Three reactions were carried out per sample. The results were expressed in the form of relative value 2^−ΔΔCt^, where ΔCt represents Ct value of the gene minus that of the internal reference gene [20], [21]. Actin gene is a widespread housekeeping gene in higher plants, highly conserved in the same family, genus. And the Actin gene has high stability in the expression level. Amplification of Actin as an internal reference was also carried out in the same sample (the primer's sequences are shown in Table 1, and the amplification product is 149 bp) [26]. DEPC-water for the replacement of template was used as a negative control. A relative standard curve was developed using 10-fold serial diluted cDNA. The standard curves were included in all runs to relate to quantitative data. The standard curve equations of Actin gene (Figure S1) and PAL (Figure S2) gene were *y* = −3.3511*x*+26.342(R^2^ = 0.9993), *y* = −3.4369*x*+39.344(R^2^ = 0.9923) respectively. The melting curves of β-actin (Figure S3) and *PAL* (Figure S4) display single peak. [22], [23]

### Statistical Analysis

As for the result of RT-QPCR, observations at the each tissue were calculated to derive the mean and standard error (SE). All data obtained from the RT-QPCR analysis were log transformed before using data analysis with SAS 8.1. Difference was highly significant at *P*<0.01.

## Results

### Isolation and characterization of DcPAL

**Figure 1:**
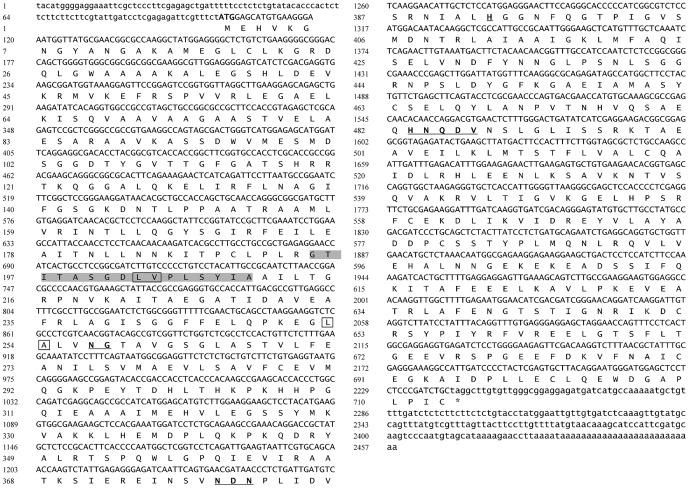
Nucleotide and deduced amino acid sequences of DcPAL. Start codon is shown in bold and italics; stop codon is indicated by an asterisk and bold fond; gene-untranslated regions are shown by standard letters; phenylalanine and histidine ammonia-lyases signature is shaded in gray; and the deamination sites are bald and in box. The catalytic active sites are bold and underlined; numbers in the left represent nucleotide and deduced amino acid sequences.

The cDNA clone was sequenced and designated DcPAL. Based on analysis using DNAStar and ORF Finder, a full-length cDNA clone was obtained using 5′/3′-RACE extension methods. Sequence analysis confirmed the clone to be a PAL gene. The full-length DcPAL comprises 2,458 bp with an open reading frame of 2,142 bp, which encodes a protein of 713 amino acid, and it also contains a 101-bp 5′UTR, a 215-bp 3′UTR, and a 26-bp polyA. The 5′ and 3′-UTRs are rich in A and T, which closely resembles the same gene from other plants. The sequence from 102 bp to 104 bp is ATG, and the nearby sequence (GTGATGG) conforms to the eukaryotic initiator codon, ATG, followed by a conserved sequence (A/GXXATGG), and there is a typical polyadenylation signal sequence (aataaa/aattaaa) at the 3′-UTR, as shown in Fig. 1. The full-length DcPAL sequence was deposited in GenBank under accession number JQ765748. Sequence analysis confirmed the clone to be a PAL gene of *Dendrobium*.

### The structure of DcPAL protein and Sequence analysis

The predicted amino acid sequence of DcPAL was highly similar to the reported plant PAL protein sequence. The DcPAL protein was further compared with homologous proteins by means of multiple sequence alignment and structure prediction analysis. The result shows that DcPAL contains the conserved PAL protein finger motif (195∼210, GTITASGDLVPLSYIA), which is a typical PAL/HAL protein tag. In addition, Blastp sequence alignment showed that DcPAL contains conserved deamination sites (i.e., L-203, V-204, L-253, and A-254) and conserved catalytic active sites (i.e., N-257, G-258, NDN [379–381 aa], H-393, and HNQDV [483–487 aa]), as shown in Figure 1.

**Figure 2:**
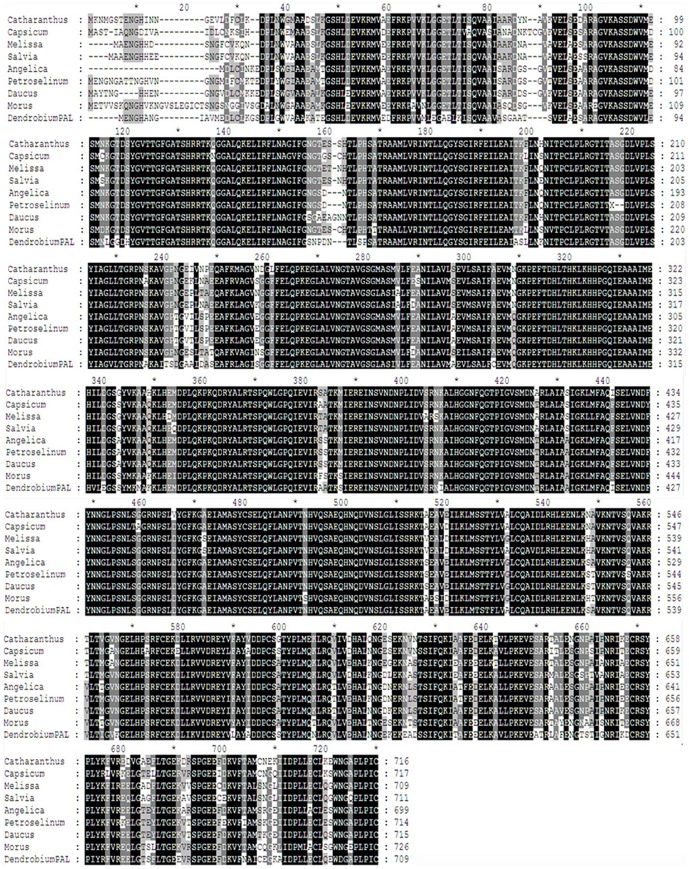
Multiple alignments of the deduced amino acid sequences of the *Dendrobium* with that of other plant species. The sequences compared are from *Jatropha curcas* (JCPAL, ABI33979.1), *Ricinus communis* (RCPAL, XP 002519521), *Populus trichocarpa* (PTPAL, XP 002326186), *Vitis vinifera* (VVPAL, XP 002268732), *Daucus carota* (DCPAL, BAC56977), *Cinnamomum osmophloeum* (COPAL, AFG26322), *Musa balbisiana* (MBPAL, BAG70992), *Phalaenopsis* × *Doritaenopsis hybrid cultivar* (PDPAL, AAP34199), *Lycoris radiate* (LRPAL, ACM61988).

These active sites are consistent with the PAL deamination site and the catalytic active sites of *Pittosporum tobira* and *Nerium oleander*. These two sites are assumed to play important roles in the function of this protein, and the presence of these sites in DcPAL suggests that DcPAL has a similar function to that of other PAL proteins. POSORT and MotifScan software analysis showed that there is a di-leucine motif LL (698–699 aa) in the C-terminal region of the DcPAL amino acid sequence, which was not found in the protein localization signal peptide at the N-terminal region. DcPAL is a soluble protein. The compute pI/MW tool was used to estimate the DcPAL amino acid sequence. This analysis revealed that the molecular mass of DcPAL is 78 kDa, with an isoelectric point of 5.78. The basic properties of the protein are similar to those of other PAL proteins. Blastp analysis indicated that the protein sequence is highly homologous to other plant PAL sequence (Figure 2), e.g., 85% sequence identity with the PAL sequence of the *Phalaenopsis* × *Doritaenopsis* hybrid cultivar, and 83% sequence identity with that of *Lycoris radiate*, 82% sequence identity with that of *Cinnamomum osmophloeum*, and 81% sequence identity with that of *Musa balbisiana*.

### Phylogenetic tree analysis

**Figure 3:**
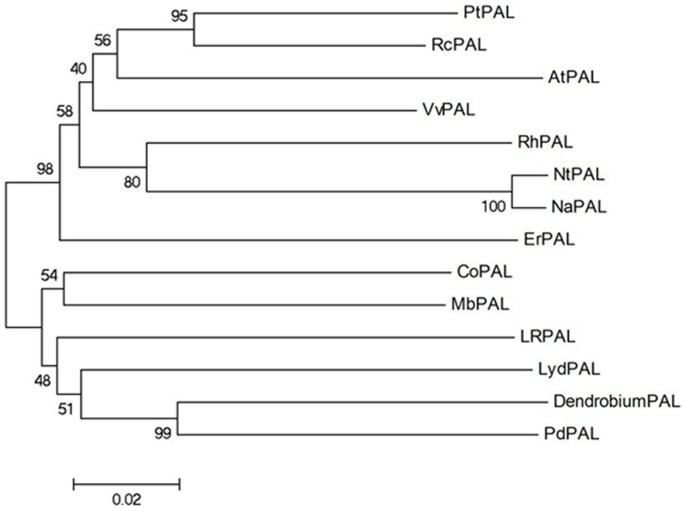
Phylogenetic tree illustrated the genetic relationships between Dendrobium PAL and other plant PALs. Sequence name and GenBank accession No. in the figure are shown as followed: *Populus trichocarpa* (PTPAL, XP 002326186), *Ricinus communis* (RCPAL, XP 002519521), *Arabidopsis thaliana* (AtPAL, NP 181241), *Vitis vinifera* (VVPAL, XP 002268732), *Rudbeckia hirta* (PhPAL, ABN79671), *Nicotiana tabacum* (NtPAL, ACJ66297), *Nicotiana attenuate* (NaPAL, ABG75911), *Eucalyptus robusta* (ErPAL, BAL49995), *Cinnamomum osmophloeum* (COPAL, AFG26322), *Musa balbisiana* (MBPAL, BAG70992), *Lycoris radiate* (LRPAL, ACM61988), *Lilium hybrid division I* (LyPAL, BAM28963), *Phalaenopsis* × *Doritaenopsis hybrid cultivar* (PDPAL, AAP34199).

Using alignments of multiple amino acid sequences, a phylogenetic tree was constructed for further identifying the relationships between the DcPAL protein sequence and that of other plants that have already been obtained. As shown in Figure 3, *Dendrobium* PAL and *Phalasenepsis* PAL appear to have similar structures and features, because *Dendrobium* PAL lined up with *Phalasenepsis* PAL.

### Phylogenetic relationship analysis

**Figure 4:**
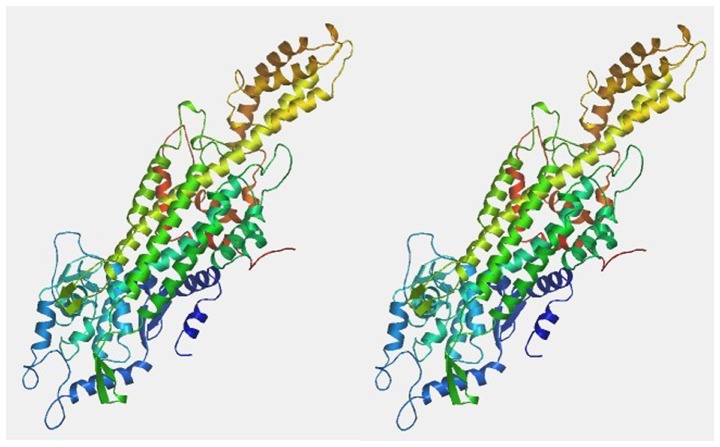
Predicted tertiary structure of DcPAL protein.

By selecting related structures after Blast analysis, which is shown in Fig.4. We found that the structure of the PAL of *Dendrobium* Sw. was very similar to that of parsley, which has a PDB ID of 1w27 and is located in the ‘B’ Chain, with a similarity level reaching 80.17%. The amino acid sequence of PAL in *Dendrobium* Sw. and the homogenous template were imported into the online tool SWISS-MODEL (http://swissmodel.expasy.org/) to further analyze the homology. The final model included residues 21–709 (E = 0.00e–1).

### Expression pattern of DcPAL

**Figure 5:**
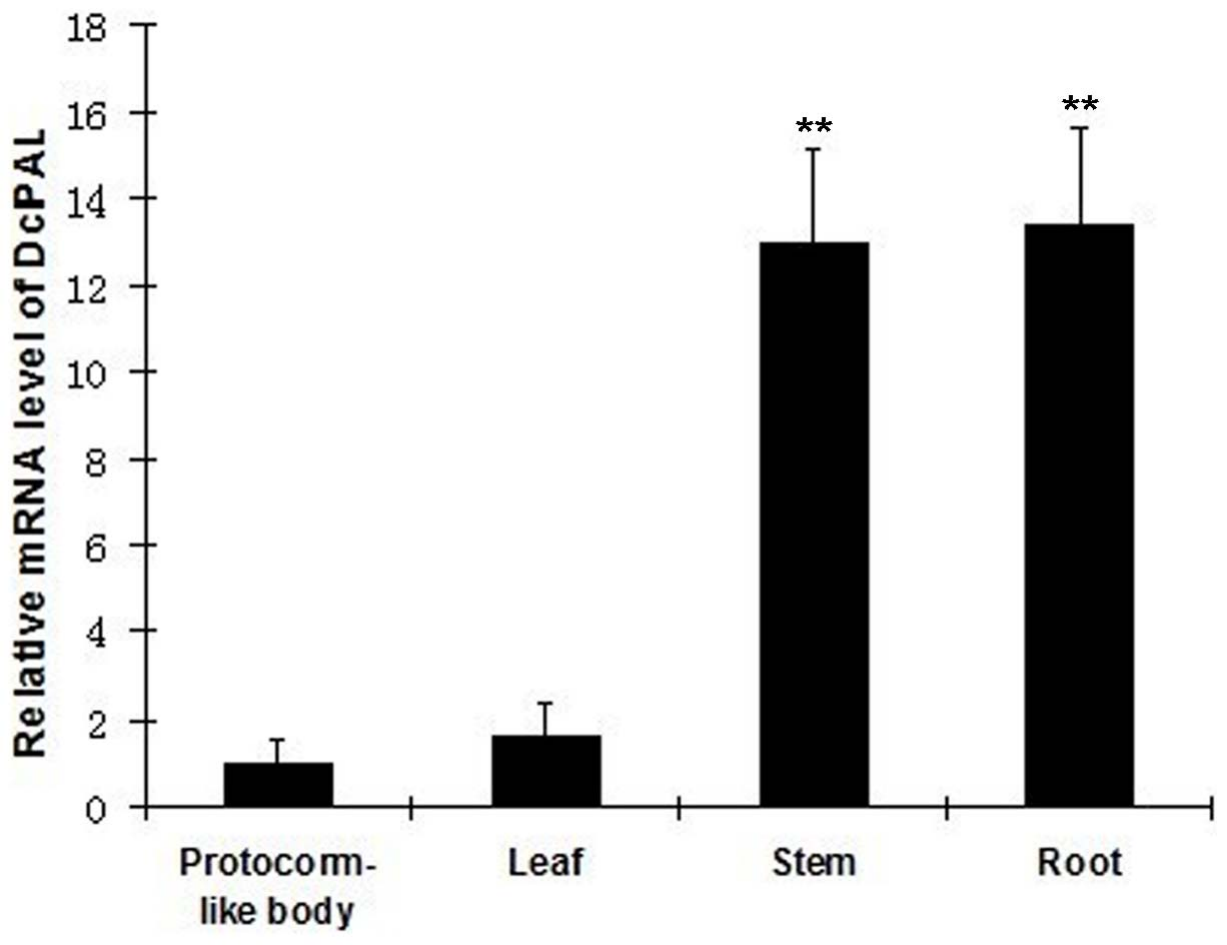
The relative expression levels of DcPAL detected in different tissues, as determined by real-time quantitative PCR. The amount of PAL mRNA was normalized to the Actin transcript level. Data are shown as means ± SE(standard error) of different tissues. The asterisks indicate significant difference by comparing with the protocorm-like bodies. The double asterisk (**) indicates the level of difference was at *P*<0.01.

For the purpose of clarifying the expression of PAL gene in each stage, we use Actin gene as the internal reference and protocorm-like bodies as the control. The DcPAL gene was found to be expressed in protocorm-like bodies, roots, stems, and leaves, as shown in Figure 5. However, the expression level varied significantly. In the protocorm-like bodies and leaves, the expression level was very low, with little difference in expression between these plant parts. However, in the roots and stems, the expression level was very high. Therefore, the influence of PAL probably varies, depending on the biological process.

## Discussion

PAL was identified several decades ago in higher plants and microorganisms[17], [24], [25]. To date, PAL genes have been identified and cloned in many different types of plants. However, no previous studies have been reported describing the PAL gene in *Dendrobium* Sw. In order to elucidate the mechanism of the biosynthesis of *Dendrobium* Sw, phenylalanine ammonia-lyase, which is the first key enzyme of phenylpopanoid pathway, was chosen for gene cloning. In this study, based on the sequence of the PAL gene in *Dendrobium candidum*, we designed specific primers and cloned PAL gene fragments from protocorm-like bodies of *Dendrobium* Sw. Furthermore, we acquired the 5′ and 3′ end sequences of this gene using the RACE method. For the first time, we obtained the full-length cDNA sequence of the PAL gene, named DcPAL, in *Dendrobium* Sw.

Two key types of sites in the DcPAL protein, including deamination sites such as L-203, V-204, L-253, and A-254 and catalytic active sites such as N-257, G-258, NDN (379–381 aa), H-393 and HNQDV (483–487 aa), are also present in PAL proteins of *Pittosporum tobira* and *Nerium indicum*, as determined using bioinformatics analysis. Furthermore, these sites are highly conserved in various plants. It was shown that DcPAL contains the conserved active-site motif (195∼210, GTITASGDLVPLSYIA) of the PAL protein, which is a typical protein tag of phenylalanine/histidine ammonia-lyase. This suggests that the predicted amino acid sequence of DcPAL is accurate. Also, several studies have concluded that homogenous sequences of the PAL gene are very highly conserved in many types of species. Thus, DcPAL is a member of the PAL gene family. The predicted amino acid sequence of the gene is highly consistent with the obtained plant PAL protein (greater than 80%). Based on multiple sequence alignments and phylogenetic analysis, *Dendrobium* PAL and *Phalasenepsis* PAL have strong connections in terms of structure and features.

Tissue expression analysis by real-time quantitative PCR revealed that DcPAL constitutively expressed in all the tested tissues, especially in stem and root. The differential expression patterns of PAL in protocorm-like body, leaf, stem and root suggest that the PAL gene performs multiple physiological functions in *Dendrobium candidum*. DcPAL is an important target in understanding the regulation of alkaloids metablism in *Dendrobium* Sw. Our current studies on DcPAL could facilitate further studies and be applied to improve alkaloids content in future *Dendrobium* Sw. cultivation.

## Supporting Information

Figure S1 **The standard curve equations of Actin gene.**
(TIF)

Figure S2 **The standard curve equations of PAL gene.**
(TIF)

Figure S3 **The melting curves of β-actin.**
(TIF)

Figure S4 **The melting curves of PAL gene.**
(TIF)
