# Supplementary figures and images for: Molecular Cloning and Sequence Analysis of a Phenylalanine Ammonia-Lyase Gene from Dendrobium
Source: PLoS One. 2013 Apr 30;8(4):e62352. doi: 10.1371/journal.pone.0062352 (PMC3640076; doi:10.1371/journal.pone.0062352)

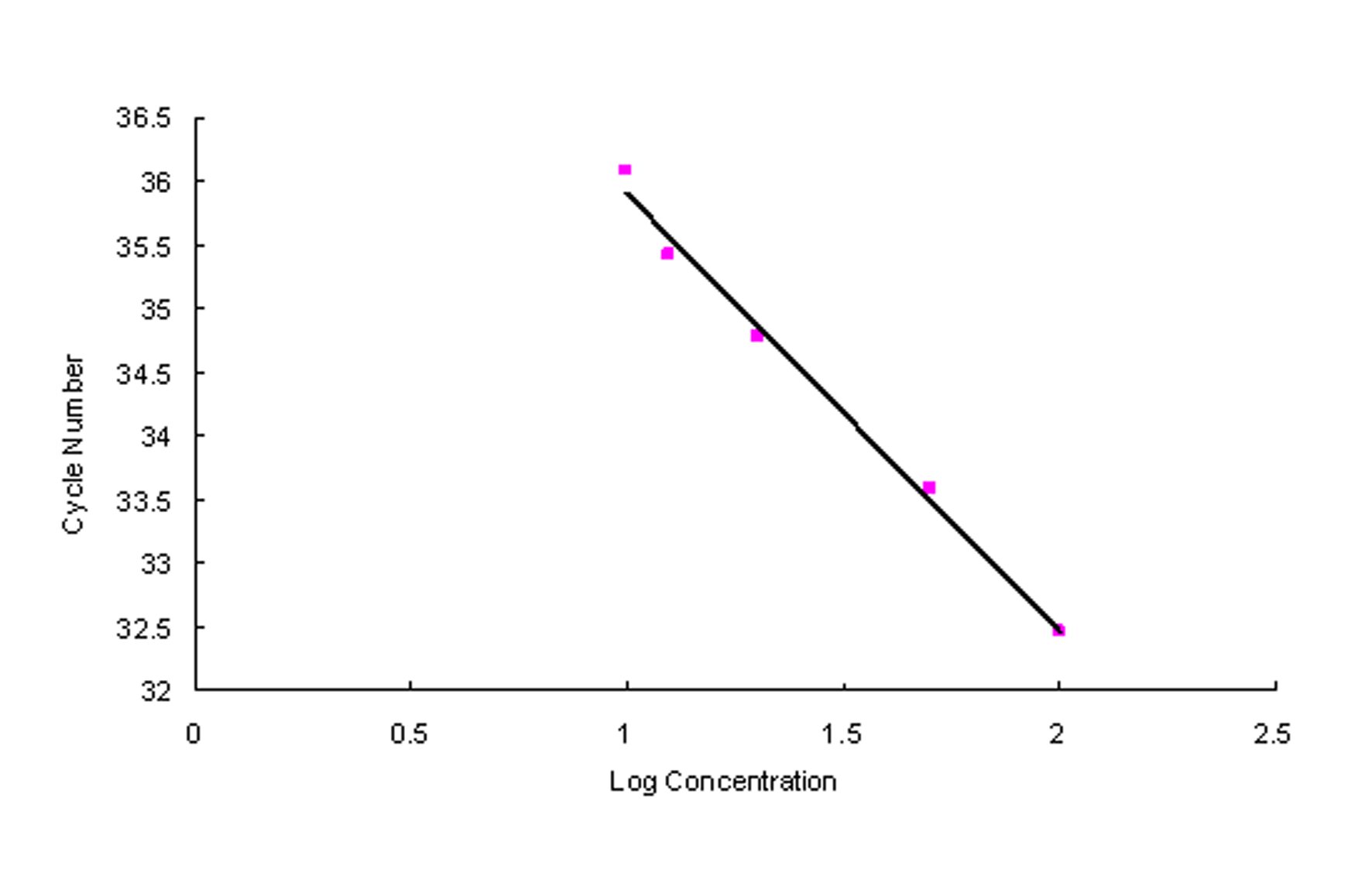

Supplement: Figure S1 — The standard curve equations of Actin gene. (TIF) [file pone.0062352.s001.tif]

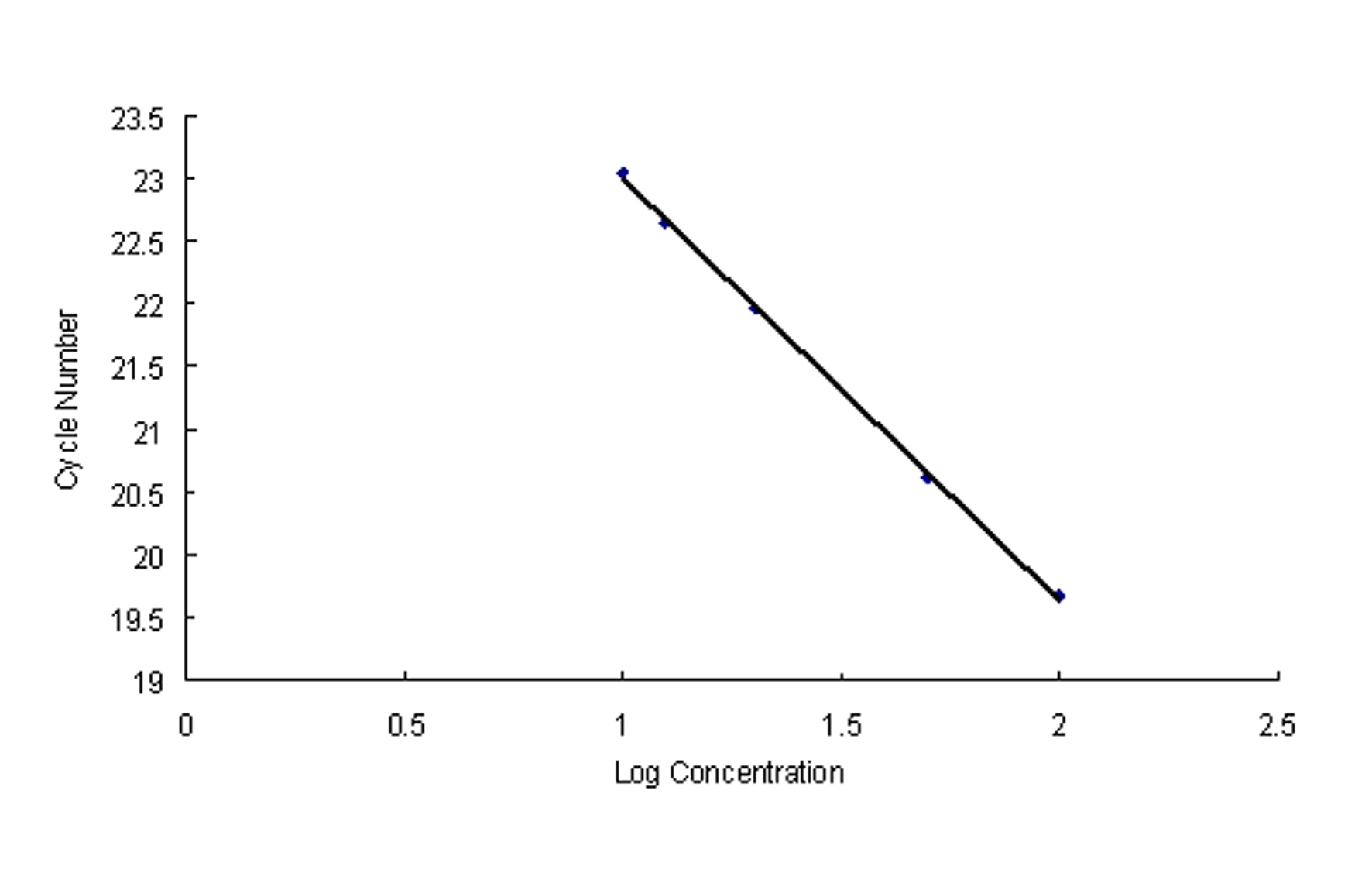

Supplement: Figure S2 — The standard curve equations of PAL gene. (TIF) [file pone.0062352.s002.tif]

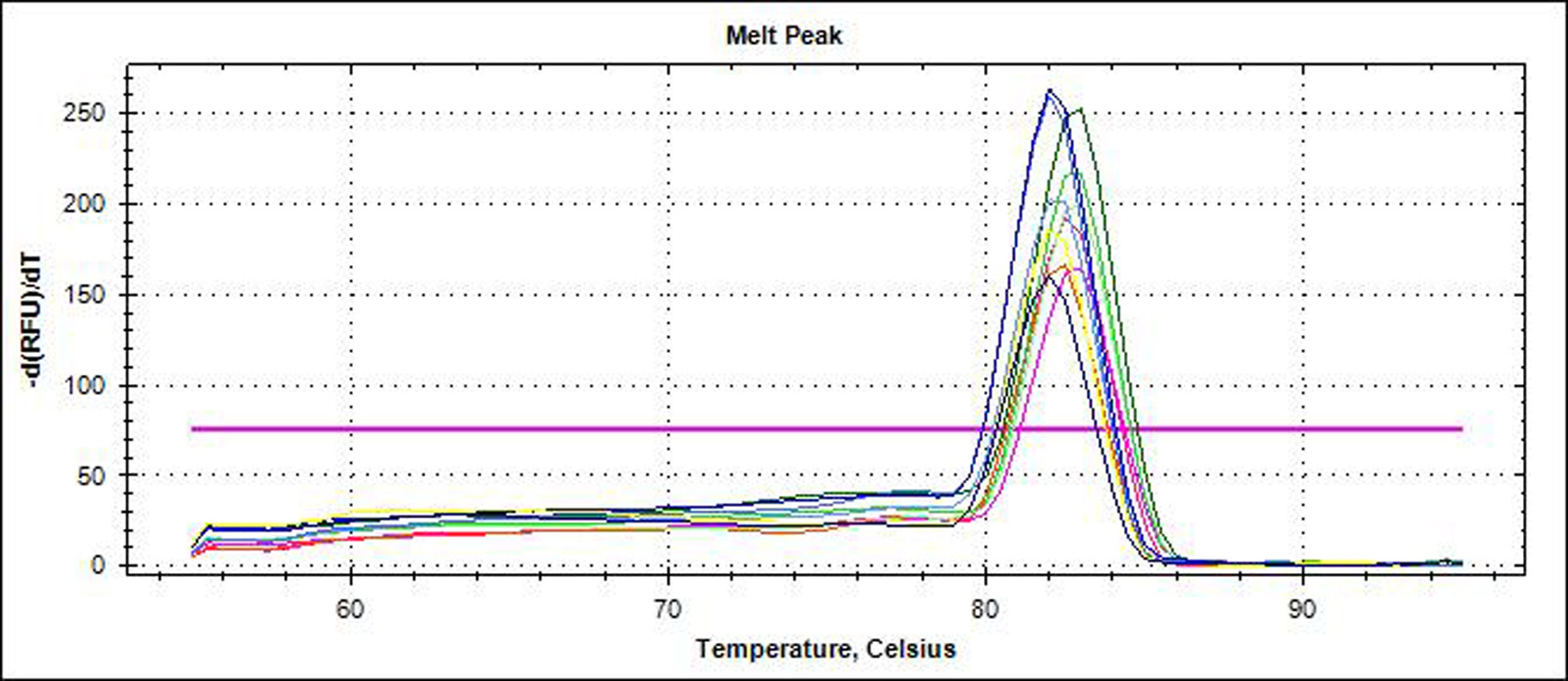

Supplement: Figure S3 — The melting curves of β-actin. (TIF) [file pone.0062352.s003.tif]

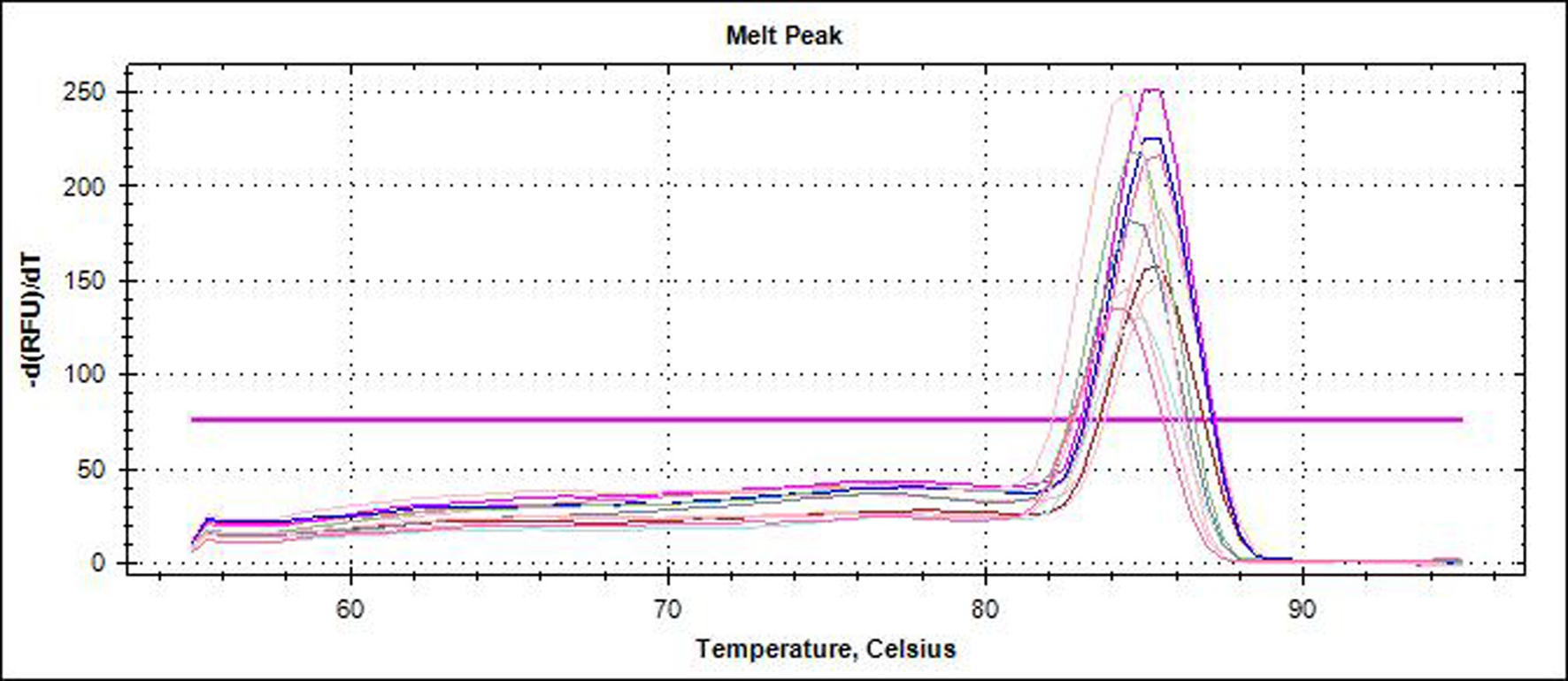

Supplement: Figure S4 — The melting curves of PAL gene. (TIF) [file pone.0062352.s004.tif]
